# The Role of Gender and Sexuality in the Experience, Internalization, and Mental Health Correlates of Sexual Victimization Stigma

**DOI:** 10.1177/08862605241246798

**Published:** 2024-04-16

**Authors:** Hilary Faithfull Farmer, Jamie E. M. Byrne, Alexander J. Mussap

**Affiliations:** 1 School of Psychology, Deakin University, Melbourne, VIC, Australia

**Keywords:** sexual victimization, stigma, shame, blame, gender, sexuality, PTSD

## Abstract

The stigma associated with sexual victimization (SV) can add to the psychological burden on survivors. We compared experiences of SV and SV stigma by survivor gender and sexuality and evaluated the relevance of public and internalized sources of this stigma to their psychological functioning. An online survey containing measures of SV type (sexual harassment and assault), public SV stigma, internalized SV stigma (self-blame, self-shame, anticipated-shame), and psychological functioning (depression, anxiety, stress, and post-traumatic stress disorder [PTSD] symptomatology) was completed by 877 women and 211 men aged 18 to 66 years (*M* = 30.2, *SD* = 8.06), of whom 73.9% were heterosexual and 26.1% identified as a sexual minority (same-sex-attracted, bisexual, pansexual, or asexual). Sexual harassment and assault were more prevalent in women and sexual minority men. Analysis of Covariance (ANCOVA) with age and SV frequency as covariates also revealed poorer psychological functioning in sexual minority men, and higher levels of SV stigma in sexual minority women and men. Multigroup path analyses further showed that exposure to public stigma was associated with poorer psychological functioning, that internalized stigma partly mediated these associations, and that the magnitude of the associations (particularly those involving self-shame and anticipated shame) was often greater in men and sexual minorities. The results add to our understanding of the role of gender and sexuality in the experience, internalization, and psychological impact of SV-related stigma on survivors. The results also highlight the need for societal shifts toward acknowledging and validating experiences of SV in men and sexual minorities, alongside women, and the development of intersectionality-informed interventions for SV stigma in survivors.

Sexual victimization (SV) refers to exposure to an actual, threatened, or attempted sexual act perpetrated without the victim’s full consent (Australian Bureau of Statistics, 2021). This is inclusive of *sexual harassment* involving unwanted sexual advances, coercive or repeated requests for sexual favors, or humiliation by exposure to offensive sexual material, and *sexual assault* involving physical contact such as unwanted kissing or touching, sexual violence, and attempted or completed rape involving penetration (Australian Government, 1984). Survivors of SV are at higher risk of poor psychological functioning, lower life satisfaction, and post-traumatic stress disorder (PTSD).

SV is understood to take place in a cultural context characterized by patriarchal gender stereotypes that disempower women and encourage them to be submissive and sexually passive, while encouraging men to be dominant, sexually confident, and initiators of sexual contact (Berry-Cabán et al., 2020).These cultural factors are implicated in gender differences in perpetration rates, with men more likely to perpetrate SV and women more likely to be victims, as well as very low reporting rates by men who have been victimized (Australian Bureau of Statistics, 2021). SV also takes place within restrictive heterosexist norms that can disempower and silence sexual minorities, increasing their vulnerability to SV and impeding their access to social protections and support (Casey et al., 2019). This may explain the elevated rates of SV reported by lesbian, gay, bisexual, and asexual (sexual minority) people (Mollet & Black, 2021).

## Public and Internalized Sources of Stigma

Cultural factors may also stigmatize survivors and add to their psychological burden (Berry-Cabán et al., 2020). Stigma describes negative attitudes or beliefs held about an individual or group that are used to devalue or justify animosity toward them (Goffman, 1986). Public stigma refers to stigma experienced from external forces, from societal messaging via the media and generally accepted norms through personal interactions with peers, organization, or services (Bos et al., 2013). Self-stigma refers to the internalization of societal attitudes toward SV survivors, resulting in feelings of self-blame and shame (Kennedy & Prock, 2018). Self-blame is a cognitive attribution of stigma in which the survivor ascribes their victimization to their own behavior or personal characteristics (Ullman et al., 2014), whereas shame is a self-conscious, painful emotional response, consisting of feelings of defectiveness, worthlessness, and a wanting to shrink or hide (DeYoung, 2015). Shame can be further divided into a feeling of disgust and dislike for the self (self-shame), and the belief that others will find them disgusting or unacceptable (anticipated shame) (Øktedalen et al., 2014). Both public and internalized stigma individually hold meaningful implications for survivors, affecting their risk of revictimization, their likelihood of help-seeking, and their psychological functioning (Kennedy et al., 2012).

## Stereotypes About Gender and Sexuality

Gender and heterosexist stereotypes are thought to underpin much of the SV-related stigma directed at survivors (De Maricourt & Burrell, 2022). Women are often held culpable for their own victimization due to societal beliefs that men cannot be held responsible for their sexual behavior (Paliliunas & Frizell, 2021). Beliefs that women use sex to manipulate men and use accusations against them to punish them (Heathy, 2020) also undermine women’s testimony in relation to SV and promote victim-blaming (Kennedy & Prock, 2018; Salim et al., 2023). Gender stereotypes about masculinity, including the belief that SV is emasculating and incompatible with heterosexuality (e.g., Hlavka, 2017; Thomas & Kopel, 2023), can also stigmatize male survivors. These stereotypes can also be internalized by men (Jannati et al., 2020), making it harder for them to recognize that they have been victimized (PettyJohn et al., 2022) and in need of help (Holliday & Monteith, 2019).

Heterosexist norms and stereotypes about sexual minorities, such as the belief interpersonal conflict between women is less serious, or that sexual minority men are highly promiscuous (Meyer, 2021), can minimize the experiences and testimony of sexual minority survivors (Thomas & Kopel, 2023). Sexual minority people are also more likely to anticipate stigma due to legitimate concerns of homophobic discrimination and being outed (Meyer, 2020), reducing their likelihood of disclosing SV (Kearns et al., 2010). SV stigma is also not limited to sexual minorities who are sexually active, with stereotypes about asexual people being at low risk of exposure to SV (despite evidence to the contrary) making coming forward about SV more difficult for them as well (Mollet & Black, 2021).

## Stigma and Psychological Functioning

The stigma encountered in response to SV disclosure has sometimes been described as akin to a “second rape” (Ahrens, 2006). This characterization is consistent with evidence of impaired psychological functioning in survivors. This includes higher rates of depression, anxiety, and stress observed in cross-sectional (Hakimi et al., 2018) and longitudinal studies (Orchowski & Gidycz, 2015) of survivors who have not been supported following disclosure (Jacques-Tiura et al., 2010).

The effects of public stigma on psychological functioning are also thought to be partly due to the internalization of this stigma, with studies in other stigmatized populations finding that internalized stigma mediated the relationship between public stigma and mental health outcomes (Stynes et al., 2022) and one article demonstrating the mediating role of shame in women sexual assault survivors (Bhuptani & Messman, 2023). Numerous studies have reported associations between self-blame and self-shame in survivors of SV and levels of depression, anxiety, and stress (Anwar et al., 2022), with longitudinal studies revealing higher rates of PTSD in the months following disclosure (Kline et al., 2021) and even several years later (Sigurvinsdottir et al., 2020).

## The Present Study

Research has investigated the pervasive nature of SV stigma and identified several reasons to suspect that this stigma will depend on a survivor’s gender and sexual orientation. Comparatively little research has been conducted into risk of exposure or psychological vulnerability to SV stigma as it pertains to men and sexual minorities (see Thomas & Kopel, 2023). Furthermore, research in the area has typically failed to partition influences from stigma that originates externally (i.e., public stigma) versus internally (i.e., internalized stigma) in this context. Therefore, in the present study, a community sample of heterosexual and sexual minority women and men who had experienced at least one instance of sexual harassment and/or assault were asked to complete an online survey that assessed frequency and type of SV, SV-related stigma, and psychological functioning (distress and PTSD symptomatology). Results were used to compare levels of public and internalized stigma as a function of gender and sexuality and assess their differential relevance to psychological functioning in these groups. Because the measures of SV stigma used in the study had not previously been validated for use in these groups, analyses were preceded by measurement evaluations. The following hypotheses were tested:

*H1*: Factor analyses will confirm the factor structure of each SV stigma scale (H1a) along with its configural, metric, and scalar invariance between gender and sexuality subgroups (H1b).
*H2*: Women and sexual minorities will report more frequent sexual harassment and sexual assault.
*H3*: Sexual minorities and men will report higher levels of SV-related stigma, along with higher levels of psychological distress and PTSD symptomatology (H3a), with an interaction between gender and sexuality expected in which sexual minority men produce relatively higher scores on these variables (H3b).
*H4*: Public SV stigma will be associated with psychological distress and PTSD (H4a), and internalized SV stigma will (partly or fully) mediate these associations (H4b).
*H5*: The associations between SV stigma and psychological functioning predicted in H4 will be larger in magnitude in men and sexual minorities.

## Method

Participants were 1,131 adults, 877 (77.5%) women and 211 (18.7%) men, between 18 and 66 years of age (*M* = 30.23, *SD* = 8.06), who reported having experienced at least one instance of sexual harassment and/or assault during their lifetime. They typically reported experiencing multiple incidents of sexual harassment (*M* = 6.17, *SD* = 2.77) and/or sexual assault (*M* = 4.42, *SD* = 2.86), with first exposure most commonly occurring in adolescence (*M*_age_ = 16.11, *SD* = 4.82). A sizeable minority of participants reported exposure to sexual harassment (*N* = 227, 24.5%) and sexual assault (*N* = 172, 25.2%) occurring as recently as the past 6 months. Most identified as heterosexual/straight (*N* = 836, 73.9%), with the rest grouped into a “sexual minority” category (*N* = 227, 20.07%) consisting of homosexual/lesbian/same-sex-attracted (*N* = 75, 6.6%), bisexual or pan sexual (*N* = 132, 11.7%), or asexual (*N* = 20, 1.8%). Participants who did not provide their sexual identity or who were non-cis (transwomen *N* *=* 6, transmen *N* = 9, nonbinary *N* = 17) were excluded from analyses due to inadequate numbers for subgroup analyses. Participants’ ethnicity/race was predominantly White (*N* = 849, 75.1%), followed by Hispanic (*N* = 56, 5%), Asian (*N* = 51, 4.5%), Black (*N* = 50, 4.4%), Jewish (*N* = 30, 2.7%), Middle-Eastern/Arabic (*N* = 25, 2.2%), multiethnic/multicultural (*N* = 23, 2.0%), Indigenous (*N* = 21,1.9%), or Pacific Islander (*N* = 15, 1.3%). Most resided in a western country such as the United States (*N* = 520, 46%%), Australia (*N* = 382, 33.78%), the United Kingdom (*N* = 29, 2.6%), or Canada (*N* = 16, 1.4%).

### Procedure

The online survey was conducted in accordance with ethical guidelines and the approval of our institution’s ethics committee. Participants were adult survivors of SV recruited from social media sites (primarily Facebook and Reddit), online support groups for SV survivors, as well as online groups dedicated to awareness-raising around SV.

### Measures

#### Demographics

Participants were asked to report their age, gender, race/ethnicity, education, employment, country of residence, yearly income bracket, and relationship status.

#### SV experiences

The Sexual Experience Questionnaire (Pinchevsky et al., 2020) was used to measure sexual assault and harassment experiences. Participants used a rating scale anchored between 0 (“never”) and 10 (“ten times”), with the option to respond 11 (“more than ten times”), to indicate lifetime frequency of experiencing each of six examples of sexual assault involving physical contact (e.g., “*Someone used physical force or threats of physical force to make you have sexual contact with them*”) and four examples of sexual harassment not involving physical contact (e.g., “*Someone made inappropriate or offensive comments about your or somebody else’s body, appearance, or sexual activities*”). This measure has been widely used to quantify SV experiences in a range of contexts and has adequate reliability and validity in men and women (Johnson et al., 2017).

#### Public stigma

The Rape Stigma Questionnaire (Jewkes et al., 2022) was used to measure public stigma experiences. Participants used Likert-type scale anchored between 0 (“somewhat disagree”) and 5 (“somewhat agree”) to respond to three items measuring different public stigma experiences: (1) “*People avoided talking to me or spending time with me due to what had happened to me*”; (2) “*I was told that I could have done more to prevent the incident from occurring*”; and (3) “*People made rude, insensitive, or inappropriate remarks to me about what had happened to me*.” This scale was modified from the South African AIDS stigma scale which has demonstrated adequate reliability and validity (Singh et al., 2011).

#### Self-blame

The Rape Attribution Questionnaire (RAQ; Frazier, 2003) was used to measure self-blame. Participants used Likert-type scales anchored between 1 (“never”) and 5 (“very often”) to respond to five items describing self-directed attributions of responsibility for SV (e.g., “*I used poor judgement*”). Responses were averaged, with higher scores indicating greater blame. The RAQ has shown good reliability and validity (Frazier, 2003).

#### Self-shame and anticipated shame

The Trauma-Related Shame Inventory-Short Form (TRSI) (Grau et al., 2022) was used to measure self and anticipated shame relating to SV experience(s). Participants used Likert-type scales anchored between 0 (“somewhat disagree”) and 5 (“somewhat agree”) to respond to five items measuring self-shame (e.g., “*I was ashamed of myself because of what happened to me*”), and five items measuring anticipated shame (e.g., “*If others knew what happened to me, they would be disgusted with me*”). Responses were averaged, with higher scores indicting greater levels of shame. The TRSI has demonstrated adequate reliability and strong validity (Grau et al., 2022).

#### Psychological distress

The Depression, Stress, and Anxiety Scale (DASS-21) (Lovibond & Lovibond, 1995) was used to measure psychological distress associated with SV. Participants used Likert-type scales anchored between 1 (“never”) and 4 (“almost always”) to respond to seven items measuring depression (e.g., “*I couldn’t seem to experience any positive feeling at all*”), seven measuring anxiety (e.g., “*I felt I was close to panic*”), and seven measuring stress (e.g., “*I found it difficult to relax*”). The DASS-21 is a widely used measure in research and clinical areas and demonstrated good reliability and validity across different ethnic groups (Norton, 2007).

#### Post-traumatic stress symptoms

The PTSD checklist for DSM-5 (PCL-5) (Ashbaugh et al., 2016) was used to measure PTSD symptomatology associated with SV. Participants used Likert-type scales anchored between 1 (“not at all”) and 5 (“extremely”) to respond to five items measuring intrusions (e.g., “*Repeated, disturbing dreams of the experience?*”), two measuring avoidance (e.g., “*Avoiding memories, thoughts, or feelings related to the experience?*”), seven measuring negative cognitions and mood (e.g., “*Trouble remembering important parts of the experience?*”), and five measuring hyperarousal (e.g., “*Trouble falling or staying asleep?*”). The PCL-5 is widely used and demonstrates strong reliability and validity within community and clinical populations (Blevins et al., 2015).

### Data Preparation and Analytic Plan

IBM™ SPSS Statistics (V29) was used to screen for missing items and confirm their randomness across measures and cases. For the purposes of preliminary measurement evaluation (see below), missing items were replaced using imputation via regression; for the purposes of subsequent inferential analyses, missing values were omitted from computation of scales. Resultant scales were screened for outliers greater than ±3.29 SD from the mean, and these were adjusted to be equal to 3.29 SD (Tabachnick & Fidell, 2007). Normality of resultant scales was evaluated in terms of skew and kurtosis and normal distribution of residuals.

Measurement invariance tests (H1) were carried out on the measures of public stigma, self-blame, and shame (shame was modeled as two correlated factors consisting of self-shame and anticipated shame) (Grau et al., 2022). These tests were conducted according to recommendations by Byrne (2010), following the reporting conventions of Putnick and Bornstein (2016), and on the basis of confirmatory factor analyses (CFA) in AMOS™ assessed against standard fit criteria: Chi-square/df ratio <5; root mean square error of approximation (RMSEA) <0.06 (with RMSEA 0.05–0.08 for fair fit), standardized root mean square residual (SRMR) <0.06, comparative fit index (CFI) >0.95, and Tucker-Lewis index (TLI) >0.95 (Browne & Cudeck, 1992; Hu & Bentler, 1999). Configural invariance was first evaluated by attempting to fit each model to each of the gender and sexuality subgroups separately. This model was then used as a “baseline model”—with both subgroups included but without equality constraints imposed—against which metric and scalar invariance could be tested. Metric invariance was evaluated by testing for significant deterioration of model fit (using a difference-of-Chi-square) when factor loadings were constrained between subgroups. Scalar invariance was evaluated by testing for additional deterioration of model fit when constraints were also imposed on intercepts. In the event of failure of invariance testing, attempts were made to identify the offending coefficient(s) by systematically releasing constraints, starting with the most-to-least discrepant, until no significant deterioration of model fit was observed (Byrne, 2010).

Differences in rates of sexual harassment and assault by gender and sexuality were tested using ANCOVAs with age as a covariate (H2), and differences in psychological functioning and SV stigma were tested using ANCOVAs with sexual harassment and assault rates included as additional covariates (H3). These subgroup comparisons were preceded by a Chi-square test of independence to confirm that membership of the gender and sexuality subgroups was not potentially confounded (this was mainly to allay concerns that men might be overrepresented in the sexual minority subgroup).

Path analyses conducted in AMOS™ using maximum likelihood estimation, with bootstrapping (2,000 resamples) to estimate *p* values (Shrout & Bolger, 2002), were used to test hypotheses concerning subgroup differences in paths from SV stigma to measures of psychological functioning (H4). The path model included direct paths from public SV stigma to DASS-21 and PCL-5, and indirect paths mediated by self-blame followed by self-shame and anticipated shame. The model was initially tested with all paths (and all correlations between variables at each step) included but was then optimized by removal of nonsignificant paths. To test hypotheses concerning subgroup differences (H5), multigroup path analyses were conducted on the optimized path model using the “manage groups” function in AMOS™. These analyses involved constraining regression weights and then intercepts and testing for significant deterioration in model fit. Follow-up analyses, in which constraints would be systematically released from most-to-least discrepant coefficients, could then be conducted to isolate the source of any disparity between subgroups.

## Results

### Measurement Evaluation of the SV Stigma Measures

CFAs on the SV stigma measurement models are summarized in Table 1. Results obtained on the combined data (the first row of fit results shown for each model) confirm adequate model fit against most fit criteria and support the use of these measures in their original/prescribed form (H1a). Fits obtained on the separate subgroups (rows containing these results are labeled “women/men” or “heterosexual/sexual minority” in the table) further demonstrate basic configural invariance for these measures, and subsequent invariance testing further confirmed adequate metric invariance between these subgroups. However, widespread failure of scalar invariance was evident in terms of higher intercepts for women in relation to self-blame, and higher intercepts for sexual minority participants in relation to public stigma, self-shame, and anticipated shame. The configural and metric invariance results supported our intention to compute scales/subscales similarly irrespective of participant gender or sexuality (H1b), but the limited scalar invariance results suggests that SV stigma experiences are more normative in women and sexual minorities and that caution should be used when comparing scores on these measures in these groups.

**Table 1.** Model Fits and Measurement Invariance Results for the Stigma-Related Measurement Models.

		Gender Invariance Tests								Sexuality Invariance Tests						
Measure	Group	χ^2^ (*df*)	χ^2^/*df*	RMSEA	SRMR	CFI	TLI	Δχ^2^ (Δ*df*)	Group	χ^2^ (*df*)	χ^2^/*df*	RMSEA	SRMR	CFI	TLI	Δχ^2^ (Δdf)
Public stigma	Combined	7.41* (1)	7.41	0.076	0.023	0.99	0.96	—		—	—	—	—	—	—	—
	Women	8.38* (1)	8.38	0.092	0.027	0.98	0.95	—	Heterosexual	0.61* (1)	0.61	0.000	0.008	1.00	0.99	—
	Men	0.03* (1)	0.03	0.000	0.004	0.99	0.99	—	Sexual minority	4.02* (1)	4.02	0.117	0.037	0.97	0.90	—
	Baseline	8.41 (2)	4.20	0.055	0.027	0.99	0.96	—	Baseline	4.64 (2)	2.32	0.036	0.008	0.99	0.98	—
	Metric	11.85 (4)	2.96	0.043	0.026	0.99	0.98	3.44 (2), *p* = .179 ns	Metric	6.71 (4)	1.68	0.025	0.011	0.99	0.99	2.06 (2), *p* = .456 ns
	Scalar	19.60 (7)	2.80	0.041	0.026	0.98	0.98	7.75 (3), *p* = .051 ns	Scalar	73.01* (7)	10.43	0.095	0.012	0.87	0.88	66.30 (3), *p* < .001
Metric and scalar invariance confirmed									Metric invariance confirmed; scalar invariance fails due to higher intercepts in sexual minorities							
Blame	Combined	31.72* (5)	6.34	0.069	0.027	0.98	0.95	—		—	—	—	—	—	—	—
	Women	29.92* (5)	5.97	0.075	0.031	0.97	0.93	—	Heterosexual	24.00* (5)	4.80	0.067	0.032	0.96	0.92	—
	Men	3.71* (5)	0.74	0.001	0.021	0.99	0.99	—	Sexual minority	29.07* (5)	5.81	0.146	0.045	0.95	0.89	—
	Baseline	33.52* (10)	3.35	0.047	0.031	0.98	0.95	—	Baseline	53.13* (10)	5.31	0.064	0.032	0.95	0.91	
	Metric	34.16* (14)	2.44	0.036	0.031	0.98	0.97	0.64 (4), *p* = .959 ns	Metric	54.79* (14)	3.91	0.052	0.033	0.06	0.94	1.66 (4), *p* = .798 ns
	Scalar	46.91* (19)	2.47	0.037	0.031	0.97	0.97	12.75 (5), *p* < .05	Scalar	61.02* (19)	3.21	0.046	0.033	0.95	0.95	6.23 (5), *p* = .284 ns
Metric invariance confirmed across genders; scalar invariance fails due to higher intercepts in women									Metric and scalar invariance confirmed							
Shame	Combined	150.91* (34)	4.44	0.055	0.023	0.98	0.98	—		—	—	—	—	—	—	—
	Women	116.09* (34)	3.41	0.052	0.023	0.98	0.98	—	Heterosexual	100.75* (34)	2.96	0.048	0.023	0.98	0.98	—
	Men	82.95* (34)	2.44	0.083	0.040	0.96	0.94	—	Sexual minority	89.01* (34)	2.62	0.085	0.034	0.97	0.95	—
		Gender Invariance Tests								Sexuality Invariance Tests						
		χ^2^ (*df*)	χ^2^/*df*	RMSEA	SRMR	CFI	TLI	Δχ^2^ (Δ*df*)		χ^2^ (*df*)	χ^2^/*df*	RMSEA	SRMR	CFI	TLI	Δχ^2^ (Δdf)
	Baseline	199.20* (68)	2.92	0.042	0.023	0.98	0.97	—	Baseline	189.92* (68)	2.79	0.041	0.023	0.979	0.97	—
	Metric	201.83* (76)	2.66	0.039	0.023	0.98	0.98	2.63 (8), *p* = .955 ns	Metric	216.49* (76)	2.85	0.042	0.026	0.975	0.97	26.57 (8), *p* < .001
	Scalar	212.44*(86)	2.47	0.037	0.023	0.98	0.98	10.61 (10), *p* = .389ns	Scalar	319.78* (83)	3.85	0.052	0.024	0.958	0.96	119.94 (10), *p* < .001
Metric and scalar invariance confirmed									Metric invariance acceptable (although two anticipated shame items had higher loadings in sexual minorities); scalar invariance fails due to higher intercepts in sexual minorities							

*Note*. Combined model = original model fit on combined dataset; Baseline model = subgroups tested simultaneously with no equality constraints imposed; Metric model = constraints applied to loadings; Scalar model = constraints applied to loadings and intercepts; RMSEA = root mean square error of approximation; SRMR = standardized root mean square residual; CFI = comparative fit index; TLI = Tucker-Lewis index.

* *p* < .05.

### Group Differences

Scales were computed and descriptive statistics are summarized in Table 2 separately for the gender and sexuality subgroups. Cronbach’s alphas demonstrate acceptable to adequate internal reliability, and correlations confirm expected relationships between SV stigma and both DASS-21 and PCL-5. Note that due to concerns with skew in the self-blame measure, subsequent inferential analyses were repeated on a square-root-transformed version of this variable to confirm that results were similar regardless of the nonnormality of this variable. A nonsignificant Chi-square test of independence also confirmed that membership of gender subgroup was not significantly associated with (i.e., was not confounded with) membership of sexuality subgroup, χ^2^(*df* = 1) = 0.01, *p* = .908 ns.

**Table 2.** (a) Pearson Bivariate Correlations by Gender (Women *N* = 877 Upper-Right Diagonal; Men *N* = 211 Lower-Left Diagonal) and Sexuality (Heterosexual *N* = 836 Upper-Right Diagonal; Sexual Minority *N* = 227 Lower-Left Diagonal), and (b) Descriptive Statistics for Each Subgroup.

(a)Correlations																			
Gender	1	2	3	4	5	6	7	8	9	Sexuality	1	2	3	4	5	6	7	8	9
1 Age		.18**	.36**	.12**	.03	.16**	.14**	.12**	.07	1		.22**	.33**	.15**	.01	.16**	.18**	.15	.08
2 Freq. harassed	.24**	—	.58**	−.07*	.22**	−.02	−.07	.41**	.35**	2	.05	—	.70**	−.16**	.18**	−.14**	−.18**	.40**	.28**
3 Freq. assaulted	.24**	.68**	—	−.23**	.21**	−.33**	−.26**	.45**	.32**	3	.32**	.29**	—	−.22**	.22**	−.32**	−.28**	.51**	.33**
4 Public stigma	−.03	.24**	.28**	—	.23**	.61**	.60**	.20**	.38**	4	.07	.36**	.22**	—	.26**	.64**	.61**	.24**	.41**
5 Self-blame	−.01	.09	.22**	.55**	—	.25**	.29**	.39**	.41**	5	−.08	.23**	.15*	.37**	—	.29**	.32**	.40**	.43**
6 Self-shame	.03	.31**	.26**	.70**	.57**	—	.73**	.21**	.41**	6	−.08	.44**	.10	.52**	.40**	—	.77**	.20**	.42**
7 Anticipated shame	−.01	.20**	.24**	.65**	.61**	.83**	—	.23**	.42**	7	.01	.32**	.25**	.54**	.48**	.70**	—	.23**	.42**
8 DASS-21	−.01	.33**	.49**	.66**	.51**	.65**	.63**	—	.81**	8	−.16*	.28**	.18**	.45**	.38**	.55**	.49**	—	.80**
9 PCL5	.07	.30**	.47**	.66**	.63**	.71**	.68**	.88**	—	9	−.05	.30**	.27**	.53**	.43**	.59**	.63**	.82**	—
(b)Descriptives																			
Heterosexual Women	Min	Max	*M*	*SD*	Sk/se	K/se	α		Heterosexual Men		Min	Max	*M*	*SD*	Sk/se	K/se	α		
1 Age	18	58	31.81	7.69	4.54	1.85				1	18	57	30.01	7.37	6.76	5.78			
2 Freq. harassed	0	11	6.82	2.26	−9.08	2.29	.68			2	0	11	4.32	2.78	2.66	−0.89	.88		
3 Freq. assaulted	0	9.83	5.49	2.77	−5.50	−4.83	.89			3	0	9.17	3.01	2.53	4.23	−0.03	.93		
4 Public stigma	1	6	3.08	1.10	7.15	1.36	.65			4	1	6	3.32	1.17	0.57	−0.75	.73		
5 Self-blame	1	5	3.43	0.65	−3.95	6.01	.55			5	1	4.8	3.06	0.82	−3.53	1.87	.78		
6 Self-shame	1	5	3.13	0.97	2.79	−3.66	.81			6	1	5	3.20	0.97	−2.46	−0.27	.89		
7 Anticipated shame	1	5	3.04	0.94	2.98	−2.96	.81			7	1	5	3.19	0.97	−3.55	0.16	.88		
8 DASS-21	2	63	35.91	9.28	−6.03	7.05	.82			8	0	62	28.98	12.29	−1.65	1.86	.94		
9 PCL5	1.05	5.00	3.27	0.56	−5.74	10.46	.79			9	1	4.90	3.00	0.79	−3.21	2.51	.95		
Sexual Minority Women	Min	Max	*M*	*SD*	Sk/se	K/se	α		Sexual Minority Men		Min	Max	*M*	*SD*	Sk/se	K/se	α		
1 Age	18	66	29.00	9.03	10.31	12.1	—			1	18	55	29.00	7.45	2.81	3.42	—		
2 Freq. harassed	0	11	6.59	2.93	−1.68	−2.61	.76			2	.25	11	5.90	3.23	0.47	−1.32	.83		
3 Freq. assaulted	0	11	3.59	2.57	3.49	−1.47	.78			3	0	9.67	4.16	2.81	0.79	−1.1	.82		
4 Public stigma	1	6	3.70	1.16	0.21	−1.18	.65			4	1	6	3.75	1.09	−1.06	0.66	.58		
5 Self-blame	1	5	3.21	1.02	−2.44	−0.99	.85			5	1	4.8	3.26	0.89	−1.98	0.60	.77		
6 Self-shame	1	5	3.90	0.99	−5.11	1.13	.88			6	1	5	3.78	0.93	−3.37	3.22	.85		
7 Anticipated shame	1	5	3.48	1.04	−3.02	−0.56	.91			7	1	5	3.54	0.84	−2.78	3.68	.80		
8 DASS-21	0	63	33.21	13.24	−0.82	−0.38	.94			8	14	61	35.05	11.38	1.05	−0.80	.91		
9 PCL5	1	4.95	3.23	.86	−1.61	−1.00	.94			9	1.9	4.75	3.42	0.64	0.02	−0.31	.89		

*Note*. Sk/se = skewness/standard error of skew; K/se = kurtosis/standard error of kurtosis; α = Cronbach’s alpha.

* *p* < .05, **p < .01.

**Figure 1.**
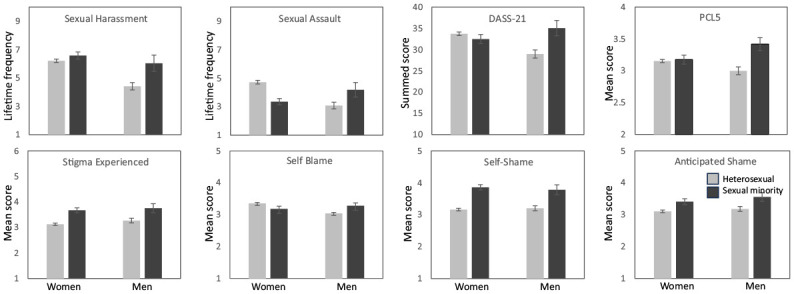
Sexual victimization and psychological functioning (top row), and stigma (bottom row) by gender (women *N* = 877, men *N* = 211) and sexuality (heterosexual *N* = 836, sexual minority *N* = 227) (±1 SE bars included).

Results of ANCOVAs by gender and sexuality (see Table 3) partially confirmed the existence of group differences in exposure to SV. As shown in Figure 1, women and sexual minorities reported significantly more sexual harassment, but not more sexual assault, than men and heterosexual participants (H3a). Sexual minority participants also reported significantly more SV-related distress (DASS-21) and PTSD symptomatology (PCL-5). These ANCOVAs also confirmed the expected interaction between gender and sexuality, with sexual minority men reporting the highest levels of SV-related distress and PTSD (H3b). An unexpected interaction effect was also observed in relation to self-blame, with straight women reporting the highest levels.

**Table 3.** ANCOVAs on Sexual Victimization Experiences and Stigma With Age as a Covariate by Gender (Women *N* = 877; Men *N* = 211) and Sexuality (Heterosexual *N* = 836; Sexual Minority *N* = 227).

Effect	Sexual Harassment *df* (1,751)			Sexual Assault *df* (1,870)			DASS-21 *df* (1,894)			PCL5 *df* (1,1026)		
	*F*	*p*	η^2^_*p*_	*F*	*p*	η^2^_*p*_	*F*	*p*	η^2^_*p*_	*F*	*p*	η^2^_*p*_
Gender	13.53	<.001	.018	0.75	.388	.001	0.31	.580	.000	0.85	.357	.001
Sex orient	14.35	<.001	.019	0.17	.682	.000	5.37	.021	.006	10.20	.001	.011
Interaction	3.91	.048	.005	16.30	<.001	.021	9.51	.002	.011	8.33	.004	.009
Effect	Public Stigma *F* (1,892)			Self-blame *F* (1,894)			Self-shame *F* (1,894)			Anticipated Shame *F* (1,893)		
	*F*	*p*	η^2^_*p*_	*F*	*p*	η^2^_*p*_	*F*	*p*	η^2^_*p*_	*F*	*p*	η^2^_*p*_
Gender	0.87	.351	.001	1.92	.167	.002	0.09	.761	.000	1.05	.306	.001
Sex orient	18.95	<.001	.021	0.13	.721	.000	38.68	<.001	.041	10.11	.002	.011
Interaction	0.05	.828	.000	6.86	.009	.008	0.23	.628	.000	0.21	.644	.000

*Note*. DASS-21 = depression, stress, and anxiety scale; PCL-5 = PTSD checklist for DSM-5.

### Multigroup Path Analyses

**Figure 2.**
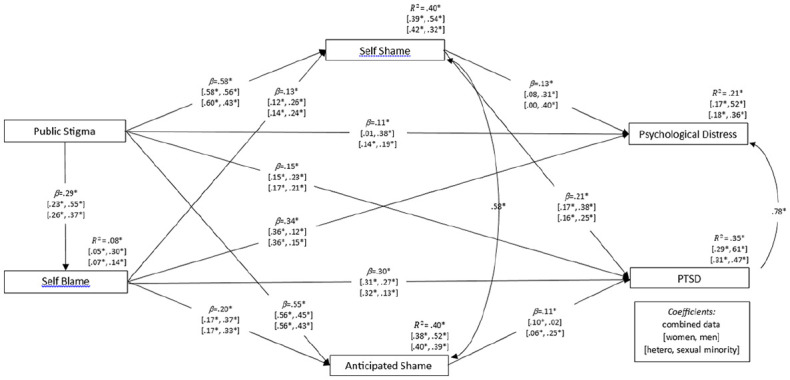
Path model used to conduct multiple mediation analysis, optimized by removal of non-significant paths. Standardized regression weights (*b*) and squared multiple correlations (*R*^2^) are presented as follows: Fit on combined data (women, men) (heterosexual, sexual minority). Correlation coefficients are shown only for combined data. Fit indices for the model fit on combined data: χ^2^ (1) = 1.46, *p* = .228 ns, χ^2^/*df* = 1.46, RMSEA = 0.020, SRMR = 0.005, CFI = 0.99, TLI = 0.99, AIC = 53.46, BCC = 53.78, ECVI = 0.05. *Note*. χ^2^ = likelihood ratio Chi-square; RMSEA = root mean square error of approximation; SRMR = standardized root mean square residual; CFI = comparative fit index; TLI = Tucker-Lewis index; AIC = Akaike information criterion; BCC = Browne-Cudeck criterion; ECVI = expected cross-validation Index. *p < .05.

The path model used to test hypotheses concerning the relationships between variables is depicted in Figure 2, fit on the combined-groups data. Removal of nonsignificant paths resulted in a model with good fit χ^2^ (1) = 1.46, *p* = .228, RMSEA = 0.020, SRMR = 0.005, CFI = 0.99, TLI = 0.99, that explained statistically significant variance in stigma-related variables: Self-blame (*R*^2^ = .08), self-shame (*R*^2^ = .40), and anticipated shame (*R*^2^ = .40), as well as SV-related psychological distress (*R*^2^ = .21) and PTSD symptomatology (*R*^2^ = .35), all at *p* < .05. The model shows direct paths from public stigma to both psychological distress and PTSD, confirming that SV stigma has a negative association with psychological functioning (H4a), and also significant indirect paths to psychological distress, β_public stigma → internalized stigma → psychological distress_ = .18, *p* < .05, and PTSD symptomatology, β_public stigma → internalized stigma → PTSD_ = .28, *p* < .05, mediated by internalized stigma. These mediations support the hypothesis that the psychological impact of public stigma is in part due to this stigma being internalized by survivors (H4b).

The model’s good fit meant that it could also be used as a baseline model from which to conduct multigroup comparisons of regression weights and intercepts. Significant discrepancies were found in relation to regression weights between gender subgroups, Δχ^2^ (Δ*df*) = 95.17 (12), *p* < .001, and sexuality subgroups, Δχ^2^ (Δ*df*) = 71.79 (12), *p* < .001. These were indicative of stronger paths from self-blame to psychological functioning in women and heterosexual participants, and stronger paths from shame (both self-shame and anticipated shame) to psychological functioning in men and sexual minority participants. Discrepancies were also found in relation to intercepts between genders, Δχ^2^ (Δ*df*) = 51.45 (12), *p* < .001, and sexualities, Δχ^2^ (Δ*df*) = 39.96 (5), *p* < .001, with consistently higher variance explained in most variables for men and sexual minorities. These results are consistent with the hypothesis that the contribution of SV stigma to psychological functioning is greater in men and sexual minorities (H5).

## Discussion

Our study was conducted to examine experiences of SV-related stigma through the lens of gender and sexuality. Before considering our findings, it is worth noting that participants’ ratings of SV exposure and mental health supported our focus on gender and sexuality. Specifically, our participant subgroups not only differed in terms of SV exposure and SV-related distress, but gender and sexuality identities also appeared to intersect to produce unique experiences of and reactions to SV. As hypothesized, sexual harassment and assault were most commonly reported by women and sexual minorities (Sundaram et al., 2008); in addition, as hypothesized, men shared in these experiences, with sexual minority men reporting particularly high rates of sexual harassment and assault, comparable to heterosexual women and even higher than sexual minority women (Thomas & Kopel, 2023). Similar results were obtained in relation to SV-related mental health. As hypothesized, although women reported the poorest mental health overall it was sexual minority men who reported the highest levels of distress and PTSD symptomatology in relation to SV experiences (Thomas & Kopel, 2023). These results do not argue against the gendered nature of SV nor its cultural origins in patriarchal systems of power and privilege (Berry-Cabán et al., 2020), but they do suggest the existence of broader and more nuanced risk factors for SV than gender alone (Mongelli et al., 2019). In doing so, these results underscore the value of including male and sexual minority perspectives in SV research, prevention, and support services (Lowe & Rogers, 2017).

### Gender, Sexuality, and SV-Related Stigma

Participants commonly encountered SV-related stigma in their public encounters with others. It was hypothesized that heterosexual men would be exposed to more public SV stigma than women. We based this prediction on gender stereotypes about SV that add emasculation to the list of harmful attitudes and beliefs specifically targeting male survivors (Jannati et al., 2020; Thomas & Kopel, 2023). However, we found no significant gender differences in relation to public stigma. The similarity between heterosexual women’s and men’s experiences of public stigma again challenges the dominant narrative of SV as an issue primarily affecting women (Thomas & Kopel, 2023). SV stigma was also internalized as self-blame and shame equally by men and women SV survivors. It was hypothesized that heterosexual men would internalize stigma more than women due to the gendered nature of interpersonal violence (Berry-Cabán et al., 2020), which may make it more difficult for men to process SV experiences. Although this hypothesis was not supported, the similarity between men’s and women’s internalization calls for a broadening of SV stigma research to include perspectives from both men and women survivors of SV (PettyJohn et al., 2022).

Hypothesized differences in SV-related stigma between sexuality subgroups were observed: Sexual minorities reported higher levels of public SV stigma and internalized SV stigma in the form of shame. This is consistent with the involvement of heterosexist attitudes and beliefs about victims of same-sex SV (Meyer, 2021) that deny, minimize, or trivialize harm experienced by sexual minority survivors, and that attribute responsibility to them for having participated in hetero-transgressive sexual behaviors (Meyer, 2021). More generally, it reinforces the idea that SV-related stigma is a source of minority stress in sexual minority people, one that warrants more empirical attention and better resourcing of SV-related prevention, education, and support services from a sexual minority perspective (Meyer, 2003).

### Stigma and Mental Health

As hypothesized, path analyses revealed that public and internalized SV stigma were associated with mental health in survivors. This replicates previous findings of an association between stigma and depression, anxiety, and stress (Anwar et al., 2022) and PTSD (Kline et al., 2021). It also supports the idea that SV stigma constitutes a significant psychological burden on survivors (Berry-Cabán et al., 2020).

We found evidence that the influence of public SV stigma was at least partially direct, that is, unmediated. This is consistent with previous research showing that negative stereotypes, attitudes, and beliefs about survivors can be hurtful, distinct from the pain caused by the SV event itself (Kennedy & Prock, 2018). Unfortunately, public SV stigma is widespread in the general community, among friends and family of survivors, and even in professionals tasked with providing justice and support to survivors (De Maricourt & Burrell, 2022).

We also observed that the direct associations involving public SV stigma were overshadowed by indirect associations mediated by internalized forms of SV stigma. Victim-blaming attitudes and beliefs about survivors—for example, that they were responsible for SV; that they did not do enough to avoid exposure to SV; that they were victims of their own efforts to use sex to manipulate others—(Kennedy & Prock, 2018; Salim et al., 2023) can be internalized as self-blame by survivors. In turn, self-blame is thought to be harmful because it prevents survivors from seeking legal recourse and mental health support for fear of being judged, doubted, or having their concerns minimized by others (Starzynski et al., 2005).

Internalized shame was particularly strongly associated with both public stigma and self-blame and mediated associations involving these variables and both distress and PTSD. Self-shame describes feelings of disgust and dislike directed inward (Øktedalen et al., 2014). As with blame, self-shame is thought to be problematic in the context of SV not only because it causes psychological harm but also because it potentially reduces survivors’ motivation to seek social and professional support (DeYoung, 2015). Anticipated shame is particularly problematic for survivors in this regard due to concerns that others will see them as unacceptable, disgusting, unreliable, and defective if they disclose SV (Øktedalen et al., 2014). These indirect effects highlight the need for SV mitigation efforts to address not only attitudes and beliefs held by perpetrators and members of society, but also those held by survivors themselves (Freyd & Smidt, 2019).

To this end, we suggest repurposing stigma-reduction interventions that have been developed and successfully trialed in response to stigma associated with various physical and mental health conditions. Public awareness campaigns and structured training programs have been shown to humanize victimized groups and challenge common misperceptions about them (Evans-Lacko et al., 2010; Watson & Corrigan, 2005), and we suggest that similar education and organizational-level approaches could be developed to address stigma directed at and experienced by SV survivors (Hedge et al., 2021). Advocacy and protest-based activities have enjoyed particular success at empowering stigmatized groups to collectively resist public stigma whenever it is encountered (Heijnders & Van Der Meij, 2006). Community-level approaches such as these could be harnessed to combat SV-related stigma and achieve social change (Ciszek, 2017) as well as address stigmatizing attitudes and beliefs that have been internalized (Eiroa-Orosa & Lomascolo, 2018) (see next section for discussion of the #METOO and #TIMESUP movements; De Maricourt & Burrell, 2022). Interventions at the intrapersonal or clinical level could take the form of professional training provided to mental health professionals to ensure that they are vigilant to signs of internalized stigma in their clients (Lanthier et al., 2023; Yanos et al., 2015). Professionals should receive training on how to equip their clients with the cognitive tools required to identify and reject this stigma themselves when it is encountered (Henderson et al., 2017).

### Stigma and Mental Health by Gender and Sexual Orientation

On the basis of previous research into men’s responses to SV (e.g., Thomas & Kopel, 2023), we hypothesized that SV stigma would be particularly relevant to men’s mental health. We found evidence of this primarily in the context of self-shame, which was more strongly relevant to SV-related psychological distress and PTSD symptomatology in men than in women. The relevance of internalized shame responses in men agrees with previous research showing that men’s distress and trauma responses to SV often feature shame and guilt in relation to what others will think of their sexuality, masculinity, and complicity in relation to the sexual act (Thomas & Kopel, 2023). Where shame was shown to be important for men’s mental health, self-blame was shown to be more important for women’s mental health. This is consistent with previous research documenting high levels of internalized blame in women (Bhuptani & Messman, 2023; Carretta & Szymanski, 2020). Susceptibility to self-blame may stem from attribution myths that feature in stereotypes about women who report SV, including their perceived responsibility for the behaviors of men and the need for them to be vigilant about their sexuality (Cowan, 2000). Internalization of this messaging may cause women to believe that they inadvertently “led on” the perpetrator, misinterpreted their actions or intentions, and/or are now potentially overreacting to what happened. Indeed, addressing gender stereotypes from a feminist perspective—by challenging feminine norms of behavior, raising awareness of the patriarchal roots of SV and SV-related stigma, and fostering a sense of empowerment and collective agency in women—has been shown to reduce self-blame in women SV survivors (Carretta & Szymanski, 2020).

As hypothesized, internalized SV stigma was more pronounced in sexual minorities and was more relevant to their psychological distress and PTSD outcomes. Sexual minorities may not have a socially acceptable framework through which to understand their SV experiences and may perceive these experiences as a personal failing and/or threat to their identity (PettyJohn et al., 2022). We suggest that this may not occur to the same extent in heterosexual women due to societal advances in acknowledging the issue of SV, as it pertains to women in a heterosexual context, such as the #METOO and #TIMESUP social media movements (De Maricourt & Burrell, 2022). Movements such as these provide a framework through which women survivors can understand the factors that conspire to blame and shame them, show them that they are not alone, have their feelings normalized and validated, and be encouraged to seek support (Sigurdardottir & Halldorsdottir, 2021).

Currently, these frameworks are less relevant for and less accessible to sexual minority and male SV survivors. This underscores the need to replace simplistic stereotypic notions of sexual perpetration and victimization with an inclusive and nuanced understanding of SV—that it stems from patriarchal and heteronormative power structures which oppress not only women but also men and sexual minorities as well (PettyJohn et al., 2022). To this end, public education around SV should ensure diverse representation of SV that challenges assumptions about what a “real” victim is (PettyJohn et al., 2022). The training of professionals who work with SV survivors should similarly include a focus on diversity, with content that directly challenges preconceptions about SV and encourages reflection about one’s own biases and assumptions (Davies, 2002; Thomas & Kopel, 2023). Finally, social movements that have arisen to provide collective resistance against patriarchal oppression should continue to ensure that they give a voice to all people affected by SV (De Maricourt & Burrell, 2022).

### Limitations

The cross-sectional research design and correlational analyses we used prevent us from drawing causal inferences from our results. The majority of our participants reported having experienced both harassment and assault, limiting our ability to differentiate between sources of stigma associated with these experiences. The effects of stigma in silencing victims are likely to have created systematic differences between survivors who were willing to participate in our study and those who were not (Freyd, 2012) and this is likely to limit generalizability of our findings to members of the former group. Although participation in the study was open to all participants who had experienced SV, most participants who did participate were White and resided in a developed western country, thus limiting generalizability of our findings to a narrow range of ethnocultural identities and nationalities. This also prevented us from testing the possibility that race/ethnicity intersects with gender and sexuality to shape survivors’ experiences of stigma (Coston, 2019). Furthermore, our sample was insufficient in size and diversity to allow us to explore cross-country differences (we encourage others to consider doing so if their sample permits). Our study also recruited insufficient numbers of non-cisgender participants to allow meaningful analyses of their experiences of SV (they were thus not included in our article). Targeted recruitment of trans and gender diverse people is required to enable proper inclusion of their experiences of SV and SV-related stigma into this growing body of literature.

Finally, our psychometric evaluations of the stigma measures confirmed invariance only in relation to the configural and metric properties of these measures. Scalar anomalies typically took the form of higher item intercepts produced by women and sexual minority men. While configural and metric invariance justified our intention to compute SV stigma scales and subscales as advocated by the authors of the measures, the failure of scalar invariance (1) calls for caution when comparing scale/subscale scores on these measures between subgroups, and (2) requires us to consider alternative explanations of the findings obtained using these measures. For example, our finding that sexual minorities, especially men, reported more SV-related stigma may be due to heterosexual women having underreported their experiences of SV stigma due to this stigma being more widespread, in other words, more “normative,” in women (Kennedy & Prock, 2018). These results, and the ongoing challenges associated with the use of these measures, highlight the need for further measurement evaluation of these measures beyond the heterosexual and undergraduate women samples that commonly feature in psychometric research in the area.

## Conclusion

Our results add to growing evidence that stigma experienced in relation to SV is psychologically harmful to survivors and differs according to the survivor’s gender and sexuality. Although stigma from public sources is implicated in this harm, our results point to negative attitudes and beliefs that have been internalized by survivors as being more problematic. Specifically, stigma internalized as shame features prominently in attitudes and beliefs held by men and sexual minority survivors and appears to be more relevant to their SV-related distress and PTSD symptomatology, whereas stigma internalized as blame features prominently in women and appears to be more relevant to their distress and PTSD symptomatology. In sum, our results underscore the need for preventative, awareness-raising, and therapeutic efforts that address stigma about SV survivors in the form of gender and sexuality-specific negative stereotypes, attitudes, beliefs, and myths. Our results also call for a nuanced approach to these efforts, one that accounts for the influence of gender and sexuality on how SV is experienced, how it is interpreted, and how others respond to it.
